# Missouri State Dental Association

**Published:** 1892-09

**Authors:** William Conrad

**Affiliations:** St. Louis, Mo.


### PROCEEDINGS.

### Missouri State Dental Association.

The twenty-seventh annual meeting of the Missouri State Dental Association was held at Clinton, Mo., July 5th to 8th inclusive. There were twenty-six new members admitted and eleven papers read. The following is the completed list of officers and committees elected for the ensuing year:

President, Dr. J. D. Patterson, Kansas City; First Vice-President, Dr. W. E. Tucker, Springfield; Second Vice-President, Dr. De Coursey Lindsley, St. Louis; Corresponding Secretary, Dr. Wm. Conrad, St. Louis; Recording Secretary, Dr. H. A. Rubey, Clinton; Treasurer, Dr. Jas. A. Price, Weston.

Executive Committee—Dr. C. B. Hewitt, Kansas City; Dr. A. J. McDonald, Kansas City; Dr. J. E. Crozier, Lees Summit.

Board of Censors—Dr. E. E. Shattuck, Kansas City; Dr. H. A. Cress, Warrensburg; Dr. E. B. Crane, California.

Committee on Ethics—Dr. Frank Slater, Rich Hill; Dr. J. B. Newby, St. Louis; Dr. C. L. Hungerford, Kansas City.

Law—Dr. Jas. A. Price, Weston.

Committee on Publication—Dr. J. E. Crozier, Lees Summit; Dr. T. J. Frey, Moberly; Dr. C. L. Hickman, St. Louis.

Committee on New Appliances—Dr. C. L. Hungerford, Kansas City.

Supervisor of Clinics—Dr. C. H. Darby, St. Joe,

The next meeting of the association will be held at Excelsior Springs, Mo., on the first Tuesday after July 4th, 1893.

WILLIAM CONRAD, Cor. Sec'y.

ST. LOUIS, MO.

